# Predicting motor recovery using surface electromyography in people with severe motor impairment after stroke: A prospective cohort study protocol^✰^

**DOI:** 10.1016/j.mex.2026.103930

**Published:** 2026-04-24

**Authors:** Farah, Vennila J, Aparna Ramakrishna Pai, Arvind N Prabhu, Sivakumar Balasubramanian, Senthil Kumaran D, Margit Alt Murphy, John M Solomon

**Affiliations:** a Department of Physiotherapy, Manipal College of Health Professions, Manipal Academy of Higher Education, Manipal, India; b Manipal College of Health Professions, Manipal Academy of Higher Education, Manipal, India; c Department of Neurology, Kasturba Medical College, Manipal Academy of Higher Education, Manipal, India; d Department of Bioengineering, Christian Medical College, Bagayam, Vellore, Tamil Nadu, India; e Department of Health & Rehabilitation, Institute of Neuroscience and Physiology, University of Gothenburg, Sweden; f Department of Clinical Neuroscience, Institute of Neuroscience and Physiology, University of Gothenburg, Sweden

**Keywords:** Cerebrovascular disease, Prognosis, Motor recovery, Functional recovery, Electromyography

## Abstract

Stroke is a leading cause of long-term disability, resulting in motor impairments that significantly affect the quality of life. Predicting motor recovery is particularly difficult for people with severe motor impairment early after stroke. Traditional assessments are limited in terms of residual muscle activity, which is crucial for tailoring rehabilitation interventions. Incorporating surface electromyography (sEMG) assessment can provide a deeper understanding of motor recovery potential in severe motor impairment. This prospective cohort study aims to develop a TRIPOD-compliant prediction model to examine whether sEMG amplitude recorded from five upper and four lower extremity muscles within the first week of stroke could predict motor recovery at 3 months post-stroke. This prospective study will include 76 participants between the ages of 18–80 years presenting first-ever anterior or middle cerebral artery stroke resulting in a severe motor impairment within the first week of stroke onset. In addition to sEMG recording to evaluate muscle activity, participants will undergo a comprehensive clinical assessment of motor functions and activity at the 1st and 3rd months following stroke onset. Multivariate regression analyses will be used to determine associations between sEMG metrics and recovery outcomes.

## Graphical abstract

**Figure:**
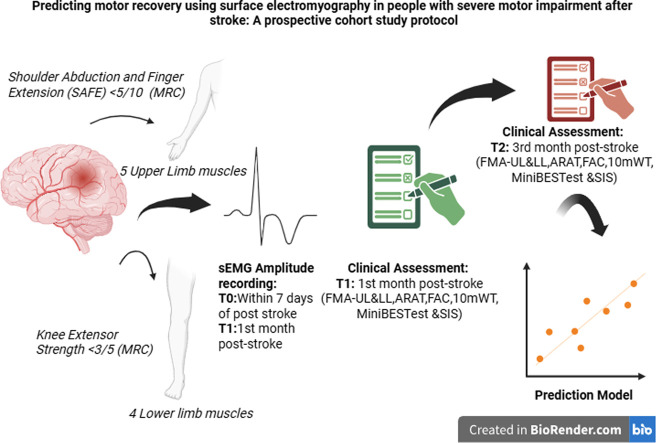

## Specifications table

**Table:** 

Subject area	Neuroscience
**More specific subject area**	Stroke
**Name of your protocol**	Predicting motor recovery using surface electromyography in people with severe motor impairment after stroke: A prospective cohort study protocol
**Reagents/tools**	• Electromyography (Delsys, Trigno wireless EMG 8-channel AD instrument, USA) • Action Research Arm Test Kit
**Experimental design**	Not applicable
**Trial registration**	CTRI/2025/06/088335
**Ethics**	Before the study commencement, all participants will provide written informed consent.
**Value of the Protocol**	• This study explores sEMG as an early biomarker of residual muscle activity, potentially enabling more accurate prediction of motor recovery in people with severe motor impairment than conventional clinical assessments alone. • By identifying patients with residual motor potential despite severe initial motor impairment, the resulting prediction model can guide individualized rehabilitation intensity and goal setting, optimizing functional outcomes. • Few studies have focused on individuals with severe motor deficits in the acute phase. This protocol generates novel evidence that could reshape prognostic practices and inform future research in this high-risk population.

## Background

Stroke is a significant contributor to death and long-term disability worldwide [1]. Poststroke motor impairment is the most prevalent cause of adult disability, which has a major impact on healthcare worldwide [[2], [3], [4]]. Such impairments limit the capacity to carry out activities of daily living (ADL) [5], with >70% of stroke survivors experiencing hemiplegia affecting the upper or lower limbs, thereby affecting their functional independence [6]. According to International Classification of Functioning, Disability and Health (ICF) [7], motor functions encompass neuromusculoskeletal and movement-related functions, including muscle strength and endurance; voluntary movement control and coordination; and movement patterns related to walking, transfers, or upper body tasks [8].

Stroke recovery, in turn, refers to the extent to which functioning at different levels (body structure/function, activity capacity, and performance or participation) has returned to a prior stroke level [9]. Recovery is a multifaceted process that possibly combines spontaneous and learning-dependent processes, including restitution (repairing the impaired neural tissues), substitution (reorganizing partially impaired neural tissues to regain lost functions), and compensation (improving the gap between a patient's impaired skills and the demands of their environment) [10]. Even though recovery trajectories differ among individuals, several studies have suggested that recovery of body functions and activities is predictable even in the first days of stroke [10,11].

The capacity to estimate motor recovery would improve health resource use, individual-tailored rehabilitation programs, and patient and therapist expectations. Nevertheless, predicting motor function recovery remains a significant challenge owing to the high degree of variability among individuals, particularly when prediction relies solely on clinical evaluation [12]. While clinical assessment can serve as a strong independent predictor, particularly for patients with mild to moderate motor impairment [13], its predictive accuracy is more limited in those with severe deficits [13]. In such cases, adding neuroimaging and neurobiology biomarkers to prognostic models may help patients with severe motor impairments [[14], [15], [16]]. Unfortunately, the high expense, technical difficulty, and restricted accessibility of these methods in standard clinical practice limit their applicability [14].

Due to the limits of clinical evaluations and the restricted availability of advanced neuroimaging technologies, alternative, affordable, and scalable approaches to enhance the prediction of motor recovery are needed, particularly in individuals with severe motor impairments after stroke. While clinical assessments are widely used, their predictive accuracy is restricted in these cases because they cannot identify subtle neuromuscular activation [17]. This limitation often results in delayed or insufficient rehabilitation strategies, a problem further compounded by the limited availability or slow clinical translation of advanced neuroimaging and neurophysiological techniques in many clinical settings. Several prognostic models have been developed to predict motor recovery after stroke, such as the Prediction Recovery Potential (PREP) and the updated version as PREP2 [12,17]. These prediction models combine early clinical assessment of Shoulder Abduction and Finger Extension score (SAFE) with neurophysiological markers such as motor evoked potentials (MEPs) obtained through transcranial magnetic stimulation (TMS) and neuroimaging measures such as diffusion-weighted Magnetic Resonance Imaging (MRI). While these approaches have demonstrated strong predictive validity, their reliance on advanced technologies limits their feasibility in routine clinical practice. This underscores the need for alternative, accessible biomarkers that can provide similar prognostic value without the cost and complexity of TMS or MRI.

In this context, surface electromyography (sEMG) may be a promising alternative. It is an objective method that offers real-time data on muscle activity in terms of timing, location, contraction intensity [[18], [19], [20]] and can also identify subtle neuromuscular signals that will not be detected during a traditional clinical examination [21]. sEMG has the benefit of being able to monitor muscle activity even in the absence of perceptible or obvious movement [22] which is especially useful for evaluating patients with severe motor impairments and could lead to earlier and more precise prognosis [23]. Although the precise neurophysiological mechanisms underlying these early sEMG signals are not fully established, existing evidence have demonstrated measurable muscle activity through sEMG during acute care in patients classified as flaccid through conventional clinical assessment [23]. This ability strongly relates to the current study's goal, which is to investigate the prognostic value of early sEMG signals in forecasting functional outcomes and motor recovery.

Despite its advantages, the application of sEMG in early stroke recovery prediction is still unexplored in clinical practice. Currently, the majority of applications focus on biofeedback or rehabilitative monitoring rather than prognostic modelling. The lack of established procedures, limited awareness among clinicians, and differences in signal interpretation may all contribute to this underutilization. Therefore, a thorough examination of the prognostic potential of sEMG is needed, especially during the acute stage of stroke. To bridge this gap, we aimed to develop a prediction model for people with severe motor impairment after stroke, employing surface electromyography to analyse whether the amplitude of muscle activity recorded from upper limb muscles such as anterior deltoid, middle deltoid, long head of biceps brachii, long head of triceps brachii and extensor carpi radialis and lower limb muscles such as tibialis anterior, medial head of gastrocnemius, rectus femoris, and biceps femoris within the first week of stroke could predict upper and lower extremity motor recovery and activity capacity of the upper extremity, postural balance, and walking at 3 months post stroke. These muscles were selected because they represent key proximal and distal muscle groups commonly involved in upper and lower extremity functions, balance and walking functions after stroke [24,25]. We hypothesized that greater sEMG amplitude recorded within the first week post-stroke, reflecting detectable voluntary muscle activity in the absence of visible movement, would be positively associated with upper and lower extremity motor recovery, upper extremity activity capacity, postural balance, and walking ability at 3 months post-stroke.

## Description of protocol

This is a prospective, single-center cohort study designed for the development and internal validation of a multivariable prognostic model, reported in accordance with the Transparent Reporting of a multivariable prediction model for Individual Prognosis or Diagnosis (TRIPOD) guidelines [26]. This longitudinal single-group cohort study will be conducted at the stroke unit and the neuromotor control unit of a tertiary care hospital in South Karnataka, India. Participants will be selected using a purposive sampling method. This study was approved by the Institutional Ethics Committee (IEC- 123/2025) and registered with the Clinical Trial Registry-India (CTRI/2025/06/088335).

## Participants

We will include individuals aged 18–80 years, of any sex, with a first-ever ischemic or haemorrhagic stroke affecting the middle cerebral artery (MCA) or anterior cerebral artery (ACA). Recruitment will occur within seven days of stroke onset, and individuals reporting a Shoulder Abduction and Finger Extension (SAFE) score of <5(0–4) or knee extensor strength of <3/5 on the Medical Research Council (MRC) strength grade within this period will be included. Only those who are medically stable and able to follow simple commands will be included. The exclusion criteria will be individuals with a history of bilateral clinical strokes; those admitted more than seven days after stroke onset; patients who had undergone surgical interventions such as craniotomy or craniectomy; and individuals with pre-existing neurological or musculoskeletal disorders affecting motor functions.

## Sample size estimation

The sample size was estimated using the following formula:

$$n={\left(Z\alpha /2\right)}^{2}\left(1-r^{2}\right)/d^{2}$$

where Zα/2 (Confidence Interval) = 95%, CI = 1.96, r (Estimated Correlation value) = 0.6, and d (Precision) = 20%.

Considering the above values, *n* = 61

Thus, with a dropout rate of 20%. *n* = 76

Hence, the final sample size of this prospective cohort study was 76 participants with stroke.

The sample size determination is guided by a widely accepted rule of thumb for prediction modelling, which recommends approximately 10 outcome events per predictor parameter [27]. Based on this principle, the available sample size is expected to support the inclusion of a small and clinically meaningful set of predictors, anticipated to be around 7 (≈7 degrees of freedom) while minimizing the risk of overfitting. Consistent with guidance by Riley et al [28] we considered a target global shrinkage factor of ≥0.90 to limit overfitting and aimed for an anticipated discrimination performance (AUC) of approximately 0.75–0.80. Furthermore, the modelling strategy will incorporate penalised regression techniques and bootstrap-based internal validation to obtain optimism-corrected estimates of discrimination and calibration, consistent with TRIPOD recommendations.

## Procedure

**Fig. 1:**
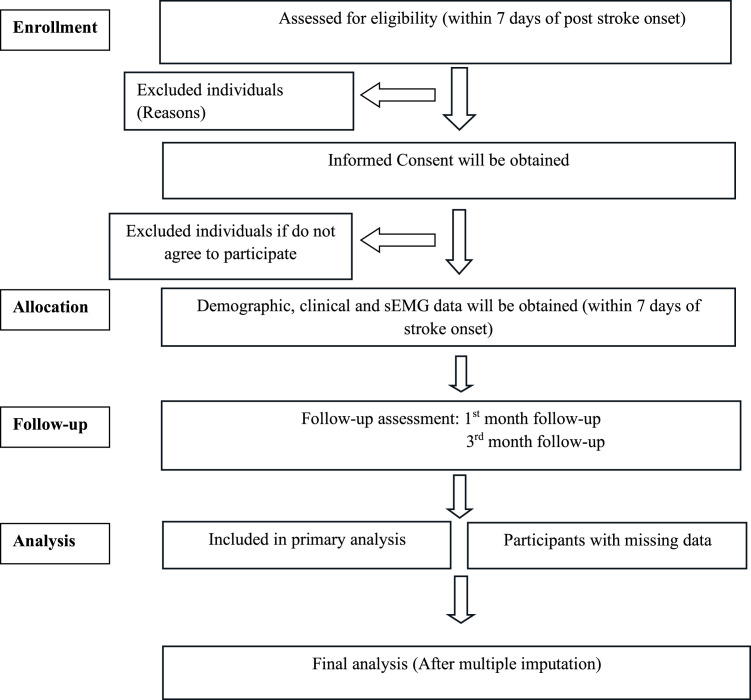
Participants flow.

All patients admitted with acute stroke to the study center during the recruitment period will be consecutively screened within the first week of stroke onset for eligibility. A screening log will be maintained to document the number of patients screened, included, excluded (with reasons), and those who decline participation. Information recorded in the screening log will inform the final participant numbers, as summarised in the study flow diagram (Fig. 1). Patients who fulfil the inclusion criteria will be requested to participate in the study. The measurements will be recorded the next day after the consent for participation. Raw sEMG signals will be collected from the paretic side at two time points—within 7 days and again at 30 days after stroke onset and processed via Lab Chart 8.1.30 software. The raw data (%Maximal Voluntary Contraction) will be normalized by the mean Root Mean Square (RMS) /max RMS X100, which will be used for analysis. Mean RMS will be computed for each 10-second trial during task execution and then averaged across trials. The following clinical assessments will be collected at the 1st and 3rd months after stroke onset: Fugl Meyer Assessment (FMA), Action Research Arm Test (ARAT), Functional Ambulation Category (FAC), 10-meter Walk Test (10MWT), Mini BEST, and Stroke Impact Scale (SIS)Version 3.0. During the hospitalization phase, all the measurements will be performed in a quiet room next to the stroke unit. During follow-up, a recording will be performed at the physiotherapy department. To minimise detection bias, assessors conducting clinical outcome assessments at the 1st and 3rd month follow-ups will be blinded to baseline sEMG measurements. Assessments will be performed by a licensed neurophysiotherapist with formal training in standardised stroke outcome measures. The investigator responsible for sEMG acquisition will be a neurophysiotherapist trained in sEMG recording, will not be involved in outcome assessments, and will remain blinded to all follow-up outcomes.

## Variable

### Independent variables (Predictor)

#### EMG recording

The upper and lower limb muscles of the affected side will be monitored via an 8-channel AD equipment from Delsys, a Trigno™ wireless EMG system (Delsys Inc., Natick, MA, USA; Model number: DSY-DS-T01D-4, 2016; Base Station ID: BID-1437; Sensor dimensions: 27 × 37 × 13 mm). The system will be operated with Lab Chart software (AD Instruments, Version 8.1.30) integrated with Trigno Control Utility (Version 3.6.0). Pre-amplifier gain and analogue bandwidth will follow the manufacturer's default settings.

To ensure correct fixation and avoid electrode and skin impedance, an alcohol-based scrub will be used to cleanse the skin. The recording area will be shaved, where necessary. The sensors will be secured to the skin with manufacturer-provided adhesives to ensure that the sensors will not fall off or move during recording. Sensors will be calibrated before each session by checking baseline noise and electrode impedance as per manufacturer guidelines. sEMG recordings at recruitment will be conducted in a quiet, separate room under standardised clinical conditions. Efforts will be made to minimise electrical interference and movement artefacts by positioning participants comfortably, avoiding proximity to high-power electrical equipment where possible, and ensuring stable wireless signal transmission.

Raw sEMG signals will be acquired at the 1000 Hz sampling frequency and preamplified (0–1.5 mv) using a Delsys electromyography system. Signals will be processed using fourth-order band-pass filtered (20–500 Hz) to reduce motion artefacts and high-frequency noise, followed by full-wave rectification and sampling at 4 kHz and then processed with root mean square (RMS) using 20-ms epochs, corresponding to the instructed movement, excluding transition periods. Crosstalk will be quantified by computing inter-channel correlation during isolated contractions, and artefact rejection will be reported as the proportion of epochs excluded due to excessive noise or signal dropout.

### Electrode placement & task instructions

**Fig. 2:**
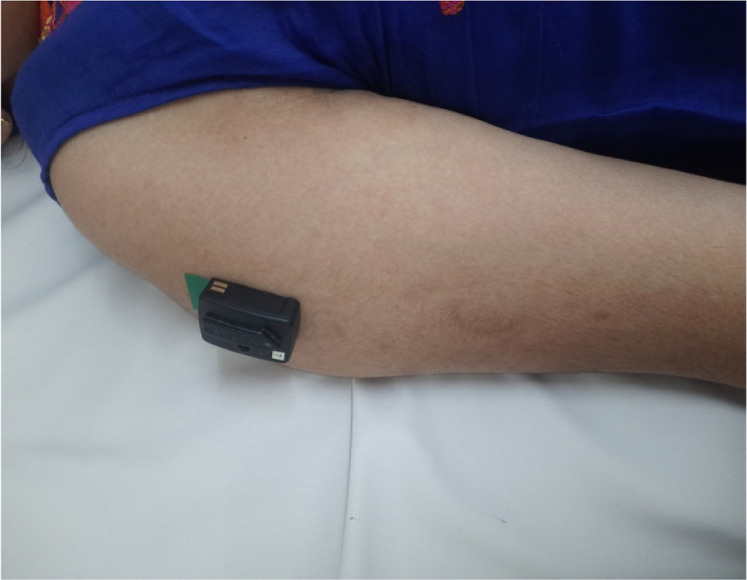
sEMG recording of middle deltoid.

**Fig. 3:**
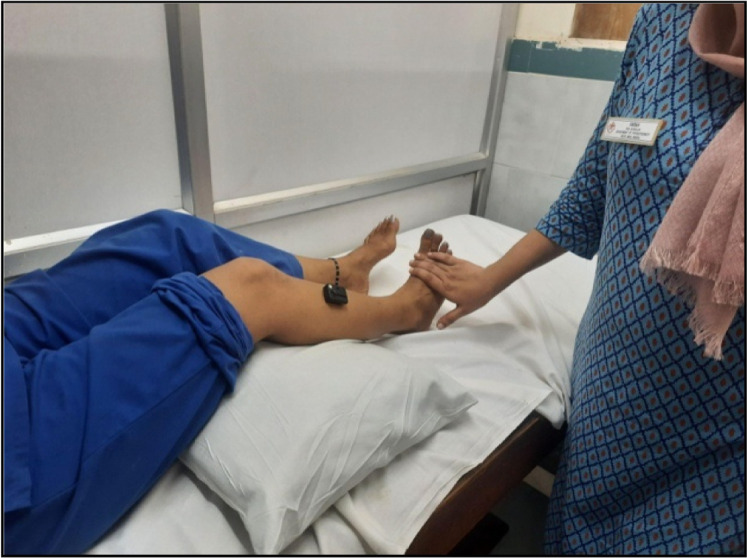
sEMG recording of tibialis anterior.

Surface EMG sensors will be positioned over the muscle belly of the affected upper and lower limb muscles in accordance with SENIAM guidelines and aligned parallel to muscle fiber orientation [29]. For sEMG recording of each muscle, participants will be instructed to perform the corresponding functional movement (eg: elbow flexion movement for the sEMG recording of the biceps brachii muscle) as depicted in Figs. 2& 3. Assistance or gentle resistance will be provided by the therapist as appropriate, depending on the participant's level of paresis. Each contraction will be recorded for a duration of 10 s, and a total of three trials will be obtained. Detailed procedures for electrode placement and recording are provided in Supplementary File 1.

### Dependent variables (outcome)

Primary outcome measures, including FMA (upper and lower motor assessments), ARAT, and FAC, along with secondary outcome measures such as 10MWT, Mini BEST, and SIS, will be assessed at the 1st and 3rd months post-stroke. The primary endpoint of this study will be motor recovery measured by the FMA score at 3 months post-stroke. The outcome will be analysed on a continuous scale, with clinically meaningful thresholds explored secondarily for dichotomous interpretation where appropriate. In accordance with SRRR guidelines [9], endpoint timing in this study is aligned to biologically informed recovery windows. The additional assessment at the 1st month captures the early subacute phase, which is a critical time point for neural plasticity [[30], [31], [32]]. In contrast, the 3-month time point provides a pragmatic and biologically credible primary endpoint for clinical trials of stroke recovery, consistent with SRRR recommendations.

The FMA is the recommended outcome of upper (FMA-UE) and lower extremity (FMA-LE) motor function in people with stroke. It assesses voluntary movement, coordination, and reflex activity in 33 upper extremity and 17 lower extremity items scored on a scale between 0 and 2 [33].

The ARAT assesses the activity capacity of the upper extremity. It has 19 items ordered in four subscales with hierarchical scoring: grip (4 items), grasp (6 items), pinch (6 items), and gross movement (3 items). ARAT is sensitive to changes in motor recovery and is frequently used both in clinical practice and research to evaluate functional outcomes of the paretic arm [34].

The FAC is a 6-point (scored as 0–5) functional walking assessment of ambulation ability and independence in walking, determining the level of human support needed, regardless of whether assistive devices are used [35].

The 10MWTat a comfortable walking speed is a reliable method for the assessment of walking speed in people with stroke [36]. The 10 MWT will only be assessed in participants able to walk independently (FAC≥3).

The Mini BEST includes evaluation of anticipatory and reactive postural control, sensory orientation, and dynamic gait. It comprises 14 items and has a maximum score of 28 points. Each item will be scored from 0–2, where 0 indicates the lowest and 2 denotes the highest level of function [37].

The Stroke Impact Scale Version 3.0 is a self-report questionnaire that assesses limitations and restrictions in activities and participation following stroke. It includes 59 items, scored on a 5-point Likert scale, evaluating strength, hand function, activities of daily living, mobility, communication, emotion, memory, and participation [38].

### Measurement time points

Independent variables (predictors) will be measured at two points: within 7 days of the first-ever stroke onset and at 1st month post-stroke. Dependent variables (outcomes) will also be assessed at two points, at the 1st and 3rd months post-stroke. (Table 1)

**Table 1:** Time points of predictor and Outcome measure assessment.

Measures		Within 7th day	1st Month	3rd Month
**Independent Variable** (Predictor)	sEMG recording of Upper limb muscles	**✔**	**✔**	
	sEMG recording of lower limb muscles	**✔**	**✔**	
**Dependent Variable** (Outcomes)	FMA-UE		**✔**	**✔**
	ARAT		**✔**	**✔**
	FMA-LE		**✔**	**✔**
	FAC		**✔**	**✔**
	10mWT		**✔**	**✔**
	Mini-BEST		**✔**	**✔**
	SIS		**✔**	**✔**

**Abbreviation:** FMA-UE, Fugl Meyer Assessment-Upper Extremity; ARAT, Action Research Arm Test; FMA-LE, Fugl Meyer Assessment-Lower Extremity; FAC, Functional Ambulation Category; 10mWT, 10-meter Walk Test; SIS, Stroke Impact Scale.

### Background variables

To describe the study population, baseline background variables, including demographic and clinical characteristics, will be recorded in accordance with core recommendations for standardized reporting in sensorimotor stroke recovery and rehabilitation trials [39].

## Quality control

### sEMG signal acquisition

Contraction tasks are standardised across participants. Visual inspection will be performed to identify artefacts related to movement, signal dropout, or excessive noise. Trials with a signal-to-noise ratio below 20 dB or excessive artefacts will be rejected. In such cases, recordings will be repeated where feasible; otherwise, affected trials will be excluded from analysis. Baseline noise levels will be checked before each recording session. Crosstalk will be minimised through SENIAM-based electrode placement, alignment with muscle fiber direction, and single-muscle task execution. All sEMG recordings will be conducted by a trained physiotherapist experienced in sEMG acquisition, following a predefined standard operating protocol, and supervised by an investigator team with over 20 years of experience in sEMG and neurorehabilitation research.

## Model development and validation

Quality control will be applied throughout model development and evaluation. Model complexity will be controlled through prespecification of predictors and the use of penalised regression to reduce overfitting and ensure consistency with the available sample size. Internal validation will be performed using bootstrap resampling to assess optimism in model performance. Evidence of substantial optimism or unstable performance will be taken as an indication of overfitting and will lead to model simplification or stronger penalisation. Model performance will be judged based on both discrimination and calibration. Poor calibration or inconsistent performance across resamples will be transparently reported. Decision-curve analysis will be used as an additional check of potential clinical usefulness**.**

## Bias

All outcome assessments will be conducted using standardized, validated methods by trained assessors to minimize measurement error and information bias. Outcome assessors will be blinded to baseline predictor data to further minimize observer bias. To reduce attrition bias, we will employ reminders and flexible scheduling to collect follow-up data at all scheduled time points for each participant. Multiple time points will be used to gather data in order to capture longitudinal changes and increase the robustness of predictive modelling.

## Statistical methods and analysis

The data will be analysed using R Studio. The normality of the distribution will be verified via the “Kolmogorov‒Smirnov test”. If the data are normally distributed, they will be presented as the mean ± SD; if they are not normally distributed, they will be presented as the median and interquartile range. Categorical variables will be presented as frequencies and percentages, or as medians and interquartile ranges. Spearman/Pearson correlation coefficients will be applied to all predictors and outcomes. Candidate predictors will be specified by using Penalised regression methods (elastic net) for model development to achieve coefficient shrinkage and reduce overfitting.

## Internal validation and model performance

Internal validation will be performed using bootstrap resampling to obtain optimism-corrected estimates of model performance. Model calibration will be evaluated by reporting the calibration intercept and calibration slope. Discrimination of the model will be assessed using the area under the receiver operating characteristic curve (AUC). Overall model performance will be quantified using the Brier score. The primary endpoint will be analysed as a (ΔFMA) as continuous outcome. Secondary analyses will explore clinically meaningful dichotomous thresholds for recovery based on published guidelines and expert consensus. Decision-curve analysis will be used to assess the net clinical benefit of the prediction model across a range of clinically relevant thresholds. In addition, repeated cross-validation using leave-one-out cross-validation (LOOCV) will be conducted as a sensitivity analysis to evaluate the robustness of model performance estimates.

## External validation

Following internal validation, external validation in an independent or temporally distinct cohort will be conducted in future work to further evaluate model generalizability and clinical utility.

## sEMG analysis

Sensitivity analyses will be conducted where feasible to assess the robustness of the primary association between early sEMG amplitude reflecting voluntary muscle activity in the absence of visible movement and subsequent motor recovery. These secondary analyses will evaluate whether reasonable variations materially influence model performance in signal-processing assumptions and will be reported as supplementary without modifying the predefined primary analytical approach. Exploratory analysis will examine the association between early sEMG amplitude and SAFE scores recorded at baseline to provide preliminary evidence of convergent validity. Spearman correlation coefficients (ρ) with 95% confidence intervals will be reported

## Uncertainty, significance and multiplicity

All key estimates will be reported, including effect sizes and 95% confidence intervals, with significant figures aligned to the measurement precision. Statistical methods will be selected based on the scale of the outcome (continuous, ordinal, or dichotomous), and model performance metrics (AUC, calibration, Brier score, and decision-curve analysis) will be reported, along with confidence intervals obtained via resampling methods. Measurement-related uncertainty arising from electrode placement, skin preparation, and normalisation procedures will be mitigated through standardised acquisition protocols, predefined quality-control thresholds, and sensitivity analyses. Multiplicity is limited by prespecifying a single primary endpoint; secondary and exploratory analyses will be interpreted cautiously, with appropriate adjustment methods applied where necessary.

## Missing data

We will make every effort to ensure completeness of follow-up data at respective time points. In cases where data is missing, multiple imputation methods will be employed to facilitate comprehensive data analysis. Missing data in predictors and outcomes will be handled using multiple imputations by chained equations (MICE), under the assumption that data are missing at random. The imputation model will include all candidate predictors, outcome variables, and relevant auxiliary variables (such as age, baseline motor severity, stroke type, and time from stroke onset to assessment) to preserve observed associations. Twenty imputed datasets will be generated, and estimates will be pooled using Rubin’s rules.

## Limitations

The study focuses specifically on patients with severe motor impairment during the first 3 months after stroke onset, which limits the generalizability of findings to broader stroke populations. Future studies could apply this protocol to patients with milder impairments, at later post-stroke stages, to improve generalizability and feasibility.

## CRediT authorship contribution statement

**Farah:** Methodology, Investigation, Writing – original draft, Visualization, Project administration. **Vennila J:** Formal analysis, Supervision. **Aparna Ramakrishna Pai:** Methodology, Validation, Resources, Writing – review & editing, Supervision. **Arvind N Prabhu:** Methodology, Validation, Resources, Writing – review & editing, Supervision. **Sivakumar Balasubramanian:** Methodology, Validation, Resources, Writing – review & editing, Supervision. **Senthil Kumaran D:** Conceptualization, Methodology, Validation, Resources, Writing – review & editing, Supervision. **Margit Alt Murphy:** Methodology, Validation, Resources, Writing – review & editing, Supervision. **John M Solomon:** Conceptualization, Methodology, Validation, Resources, Writing – review & editing, Supervision.

## Declaration of competing interest

The authors declare that they have no known competing financial interests or personal relationships that could have appeared to influence the work reported in this paper.

## Data Availability

No data was used for the research described in the article.

## Appendix Supplementary materials

Supplementary material *and/or* additional information [OPTIONAL] The detailed procedure for sensor placement and EMG recording is provided in Supplementary File 1 Image, application 1

Image, application 2
