# Supplementary material for: Predicting motor recovery using surface electromyography in people with severe motor impairment after stroke: A prospective cohort study protocol
Source: MethodsX. 2026 Apr 24;16:103930. doi: 10.1016/j.mex.2026.103930 (PMC13141797; doi:10.1016/j.mex.2026.103930)
Supplement: Supplementary file 1 — Supplementary material and/or additional information [OPTIONAL] The detailed procedure for sensor placement and EMG recording is provided in Supplementary File 1 [file mmc1.docx]

**Electrode Placement and Recording Procedure**

**Upper limb muscles-**

**1. Middle Deltoid-**

**Sensor placement-**Recording sensors will be placed over the motor point of the middle deltoid muscle from the acromion to the lateral elbow via aTrigno sensor adhesive.The positioning of the sensors will be parallel to the direction of the muscle fiber arrangement as in the direction of the line between the acromion and hand in reference to SENIAM guidelines.

**Participant Position-** Participants will be positioned in a supine lying position.

**Instructions-** To measure the peak amplitude objectively, the participants will be instructed to abduct their shoulders as much as possible against the therapist's hand resistance (MVC).

**2. Anterior Deltoid-**

**Sensor placement-**Recording sensors will be placed over the motor point of the anterior deltoid muscle at one finger width distal and anterior to the acromion via aTrigno sensor adhesive. The positioning of the sensors will be parallel to the direction of the muscle fiber arrangement in the direction of the line between the acromion and the thumb in reference to SENIAM guidelines

**Participant Position-** The participant will be positioned in a supine lying position.

**Instructions-**Tomeasure the peak amplitude objectively, the participants will be instructed to flex their shoulders as much as possible against the therapist's hand resistance (MVC).

**3. The long head of the triceps brachii-**

**Sensor placement:** Recording sensors will be placed over themotor point ofthe long head of the triceps brachii muscle muscle at 50% on the line between the posterior crista of the acromion and the olecranon at 2 finger widths medial to the line via aTrigno sensor adhesive. The positioning of the sensors will be parallel to the direction of the muscle fiber arrangement between the posterior crista of the acromion and the olecranon in reference to SENIAM guidelines

**Participant Position-** The participant will be positioned in a side-lying position.

**Instructions-** To measure the peak amplitude objectively, the participants will be instructed to extend their elbows as much as possible against the therapist's hand resistance (MVC).

**4.The long head of the biceps brachii-**

**Sensor placement-**Recording sensors will be placed over the long head of the motor point of the biceps brachii muscle muscle on the line between the medial acromion and fossa cubit at ⅓ from fossa cubit via aTrigno sensor adhesive. The positioning of the sensors will be parallel to the direction of the muscle fiber arrangement between the posterior crista of the acromion and the olecranon in reference to SENIAM guidelines.

**Participant Position-** The participant will be positioned in a supine-lying position.

**Instructions-** To measure the peak amplitude objectively, the participants will be instructed to flex their elbows as much as possible against the therapist's hand resistance (MVC).

**5. Extensor Carpi Radialis -**

**Sensor placement-**Recording sensors will be placed over themotor point ofthe extensor carpi radialis musclevia aTrigno sensor adhesive, according to SENIAM recommendations.The positioning of the sensors will be parallel to the direction of the muscle fiber arrangement in reference to SENIAM guidelines.

**Participant Position-** The participant will be positioned in a supine-lying position.

**Instructions**- To measure the peak amplitude objectively, the participants were instructed to extend their wrists as much as possible against the therapist's hand resistance (MVC).

**Lower Limb Muscles-**

**1. Rectus Femoris-**

**Sensor placement-**Recording sensors will be placed over the motor point of the rectus femoris muscle 50% on the line from the anterior spina iliaca superior (ASIS) to the superior part of the patella via aTrigno sensor adhesive., The positioning of the sensors will be parallel to the direction of the muscle fiber arrangement in the direction of from the anterior spine illiaca superior to the superior part of the patella in reference to SENIAM guidelines.

**Participant Position-** The participant will be positioned in a supine-lying position.

Instructions- To measure the peak amplitude objectively, the participant will be instructed to flex their hips as much as possible against the therapist's hand resistance (MVC).

**2. Biceps femoris-**

**Sensor placement-**Recording sensors will be placed over the motor point of the biceps femoris muscle at 50% on the line between the ischial tuberosity and the lateral epicondyle of the tibia via aTrigno sensor adhesive. , The positioning of the sensors will be parallel to the direction of the muscle fiber arrangement of the line between the ischial tuberosity and the lateral epicondyle of the tibia in reference to SENIAM guidelines.

**Participant Position-** The participants will be positioned in a side-lying position.

**Instructions-** To measure the peak amplitude objectively, the participant will be instructed to flex their hips as much as possible against the therapist's hand resistance (MVC).

**3. Tibialis Anterior -**

**Sensor placement:**Recording sensors will be placed over the motor point of the tibialis anterior muscle at ⅓ on the line between the tip of the fibula and the tip of the medial malleolus via aTrigno sensor adhesive. The positioning of the sensors will be parallel to the direction of the muscle fiber arrangement in the direction of the line between the tip of the fibula and the tip of the medial malleolus in reference to SENIAM guidelines.**Participant Position-** The participant will be placed in a supine position.

**Instructions-** To measure the peak amplitude objectively, the participant will be instructed to dorsiflex their ankles as much as possible against the therapist's hand resistance (MVC).

**4. The medial head of the gastrocnemius-**

**Sensor placement-**Recording sensors will be placed over the motor point of the medial head of the gastrocnemius musclevia aTrigno sensor adhesive, according to SENIAM recommendations. The positioning of the sensors will be parallel to the direction of the muscle fibre arrangement in reference to SENIAM guidelines.

**Participant Position-** The participant will be positioned in a side-lying position.

**Instructions-** To measure the peak amplitude objectively, the participants will be instructed to plantarflex their ankles as much as possible against the therapist's hand resistance (MVC).

**Precaution-**

Precautions will be taken for skin infections when the sEMG recording sensor is placed.
