# Supplementary material for: Predicting motor recovery using surface electromyography in people with severe motor impairment after stroke: A prospective cohort study protocol
Source: MethodsX. 2026 Apr 24;16:103930. doi: 10.1016/j.mex.2026.103930 (PMC13141797; doi:10.1016/j.mex.2026.103930)
Supplement: Supplementary file 2 [file mmc2.docx]

**Table: Model performance summary**

| **Performance metrics** | **Training dataset** | **Validation dataset**  **(Bootstrap)** | **Optimism** |
| --- | --- | --- | --- |
| AUC |  |  |  |
| Brier score |  |  |  |
| Calibration intercept |  |  |  |
| Calibration slope |  |  |  |
| Decision curve analysis (Net benefit) |  |  |  |
